# *TP53* mutation variant allele frequency is a potential predictor for clinical outcome of patients with lower-risk myelodysplastic syndromes

**DOI:** 10.18632/oncotarget.9200

**Published:** 2016-05-06

**Authors:** Monika Belickova, Jitka Vesela, Anna Jonasova, Barbora Pejsova, Hana Votavova, Michaela Dostalova Merkerova, Zuzana Zemanova, Jana Brezinova, Dana Mikulenkova, Marie Lauermannova, Jan Valka, Kyra Michalova, Radana Neuwirtova, Jaroslav Cermak

**Affiliations:** ^1^ Institute of Hematology and Blood Transfusion, Prague, Czech Republic; ^2^ First Department of Medicine, General University Hospital and First Faculty of Medicine, Charles University, Prague, Czech Republic; ^3^ Center of Oncocytogenetics, General University Hospital and First Faculty of Medicine, Charles University, Prague, Czech Republic

**Keywords:** myelodysplastic syndrome, mutational status, TP53, prognosis, variant allele frequency

## Abstract

*TP53* mutations are frequently detected in patients with higher-risk myelodysplastic syndromes (MDS); however, the clinical impact of these mutations on the disease course of patients with lower-risk MDS is unclear. In this study of 154 lower-risk MDS patients, *TP53* mutations were identified in 13% of patients, with prevalence in patients with del(5q) (23.6%) compared to non-del(5q) (3.8%). Two-thirds of the mutations were detected at the time of diagnosis, and one-third were detected during the course of the disease. Multivariate analysis demonstrated that a *TP53* mutation was the strongest independent prognostic factor for overall survival (OS) (HR: 4.39) and progression-free survival (PFS) (HR: 3.74). Evaluation of OS determined a *TP53* variant allele frequency (VAF) threshold of 6% as an optimal cut-off for patient stratification. The median OS was 43.5 months in patients with mutations detected at the time of diagnosis and a mutational burden of > 6% VAF compared to 138 months (HR 12.2; *p* = 0.003) in patients without mutations; similarly, the median PFS was 20.2 months versus 116.6 months (HR 79.5; *p* < 0.0001). In contrast, patients with a mutational burden of < 6% VAF were stable for long periods without progression and had no significant impact on PFS or OS. Additionally, we found a high correlation in the mutational data from cells of the peripheral blood and those of the bone marrow, indicating that peripheral blood is a reliable source for mutation monitoring. Our results indicate that the clinical impact of *TP53* mutations in lower-risk MDS patients depends on the level of mutational burden.

## INTRODUCTION

Myelodysplastic syndromes (MDS) are hematological malignancies characterized by ineffective hematopoiesis in one or more cell lineages, myelodysplasia and an increased risk of progression to acute myeloid leukemia (AML) [1–3]. Currently, the International Prognostic Scoring System (IPSS) remains the most commonly used system for assessing the prognosis of primary untreated adult patients with MDS [4]. The IPSS is based on the number of cytopenias, the cytogenetic profile, and the percentage of blasts in the bone marrow (BM) and stratifies patients with MDS into one of four prognostic categories: low risk, intermediate 1 risk, intermediate 2 risk, and high risk. A recently revised IPSS, the IPSS-R classification, now includes five major prognostic categories [5]. Lower-risk MDS are defined by the IPSS as low or intermediate 1 risk MDS with a lower risk of AML progression and longer survival. However, a subset of lower-risk patients shows a more aggressive disease course and shorter overall survival (OS) [6, 7]. Identification of these patients is important for risk prediction and the choice of the optimal therapeutic approach. Therefore, the identification of new prognostic factors, such as mutations in relevant driver genes, is warranted.

Protein p53 is a tumor suppressor and a transcription factor that responds to DNA damage by regulating various pathways, such as apoptosis, DNA repair, senescence and cell-cycle arrest. Somatic mutations in the *TP53* gene are one of the most common alterations in human cancers. *TP53* mutations in MDS have been described mostly in higher-risk groups, and they are associated with a complex karyotype and therapy-related MDS [8, 9]. The incidence of *TP53* mutations in lower-risk MDS patients has been evaluated in several studies: 2% in lower-risk MDS patients, as described by Bejar [10], 3% in lower-risk MDS patients and 19% in MDS patients with isolated del(5q), as reported by Kulasekararaj [11], and 18% in low-risk MDS patients with del(5q) in a study by Jädersten et al. [12].

Previous studies have suggested that *TP53* mutations were associated with worse OS and progression-free survival (PFS) [12–14] and might play an important adverse role in the malignant transformation of MDS to AML [15–17]. These mutations are found mainly in MDS patients with advanced disease, a complex karyotype, chromosome 17 abnormalities and del(5q) [10, 11]. However, the incidence and detailed effects of *TP53* mutations in a large cohort of patients exclusively with lower-risk MDS have not been analyzed using a highly sensitive technique. Examination of the mutational status of the *TP53* gene is particularly important for lower-risk MDS patients because it may significantly affect therapy decision-making. Using sensitive amplicon deep sequencing to analyze serial samples, we determined the incidence of *TP53* gene mutations in lower-risk MDS patients, the effect of *TP53* mutations on OS and PFS, the impact of treatment on mutational burden, and the level of the mutational burden with regard to the typeof cell population.

## RESULTS

### Patient characteristics

The study cohort included 154 patients with lower-risk MDS (patient characteristics are listed in Table 1). According to the WHO 2008 classification, 6 patients had refractory anemia (RA), 98 had refractory cytopenia with multilineage dysplasia (RCMD), 38 had MDS with isolated del(5q), 6 had refractory anemia with excess blasts-1 (RAEB-1), and 6 had RA with ring sideroblasts (RA-RS). All patients were low-risk (N = 70) or intermediate 1-risk (N = 81) according to IPSS. Three patients were not classified due to unavailable cytogenetic data. The median age of patients carrying mutation was 67 years (range: 50–79 years) and those without mutations was 68 years (range: 22–85 years). 2 out of 154 patients had secondary MDS who were previously treated with chemotherapy.

**Table 1:** Baseline characteristics of the patients according to *TP53* mutational status

	All (%)	Patients without mutations (%)	Patients with mutations (%)	*P* value^*^
**Number of patients**	154	134	20	
**Gender**				0.35
Male	68 (44)	61 (46)	7 (35)	
Female	86 (56)	73 (54)	13 (65)	
**Age, median (range)**	68	68 (22–85)	67 (50–79)	0.92
**Blood counts at the time of investigation**				
Hemoglobin, mean (g/l)	92.3	91.9	95.1	0.39
Neutrophils, mean (g/l)	2.8	3	1.6	0.34
Platelets, mean (109/l)	243.8	247.6	217.8	0.65
**Marrow blasts (%)**	2.5	2.4	3.2	0.11
**WHO classification 2008**				0.22
RA	6 (4)	6 (4)	0 (0)	
RA-RS	6 (4)	6 (4)	0 (0)	
MDS with isolated del (5q)	38 (25)	29 (22)	9 (45)	
RCMD	92 (60)	83 (62)	9 (45)	
RCMD-RS	6 (4)	5 (4)	1 (5)	
RAEB1	6 (4)	5 (4)	1 (5)	
**IPSS**				0.06
Low	70 (45)	65 (49)	5 (25)	
Intermediate-1	81 (53)	66 (49)	15 (75)	
NA	3 (2)	3 (2)	0 (0)	
**IPSS-R**				0.99
Very low	25 (16)	22 (16)	3 (15)	
Low	76 (49)	66 (49)	10 (50)	
Intermediate	43 (28)	37 (28)	6 (30)	
High	7 (5)	6 (4)	1 (5)	
NA	3 (2)	3 (2)	0 (0)	
**Karyotype**				0.01
Normal	53 (34)	51 (38)	2 (10)	
Abnormal	98 (64)	80 (60)	18 (90)	
NA	3 (2)	3 (2)	0 (0)	
**Karyotype by del(5q)**				< 0.001
Without del(5q)	79 (51)	76 (57)	3 (15)	
Del(5q)	72 (47)	55 (41)	17 (85)	
NA	3 (2)	3 (2)	0 (0)	
**Outcome**				
Leukemic transformation	36 (23)	27 (20)	9 (45)	
Died	53 (34)	41 (31)	12 (60)	
**Survival median (months)**	116.6	138.0	80.9	0.09
**Treatment**				
Lenalidomide	28 (18)	19 (14)	9 (45)	
5-azacytidine	8 (5)	6 (4)	2 (10)	
LEN + AZA	2 (1)	0 (0)	2 (10)	
HSCT	11 (7)	10 (7)	1 (5)	

* Comparison between patients without mutations and those with mutations. Bold font indicates statistically significant P values.

Abbreviations: RA, refractory anemia; RARS, refractory anemia with ringed sideroblasts; RCMD, refractory cytopenia with multilineage dysplasia; RCMD-RS, refractory cytopenia with multilineage dysplasia and ringed sideroblasts; RAEB-1, refractory anemia with excess blasts-1, LEN – lenalidomide, AZA – 5-azacytidine, HSCT-hematopoietic stem cell transplantation, NA: Not Available

### Cytogenetics reveals one third of normal karyotype

Conventional cytogenetics (G-banding with Wright-Giemsa stain) was performed on unstimulated culture of bone marrow cells. At least 200 interphase nuclei of BM were evaluated by fluorescence *in situ* hybridization (FISH) and complex karyotypes were analyzed using mFISH and mBAND methods. Cytogenetic analysis revealed a normal karyotype in 53 (34.4%) patients and an abnormal karyotype in 98 (63.6%) patients; 3 (1.9%) patients had unavailable cytogenetics. A total of 72 (46.8%) patients carried del(5q). A complex karyotype (including a reciprocal translocation between chromosomes 7 and 17) was found in one patient with an allele frequency of this mutation > 99%; uniparental disomy of 17p was found in one patient with an allele frequency of 62%.

### *TP53* mutations identified in 1/8 lower-risk MDS and 1/4 5q- patients

We initially sequenced samples from 154 patients (105 BM mononuclear cells, 35 BM granulocytes, 8 whole BM cells, and 6 peripheral blood (PB) granulocytes) at an average of 32.1 months from diagnosis (range: 0–131 months) using amplicon deep sequencing of *TP53* mutations on a Roche 454 GS Junior system. If a mutation was detected, a different cell population and all previous (including the time of diagnosis) and subsequently available samples were sequenced.

**Figure 1:**
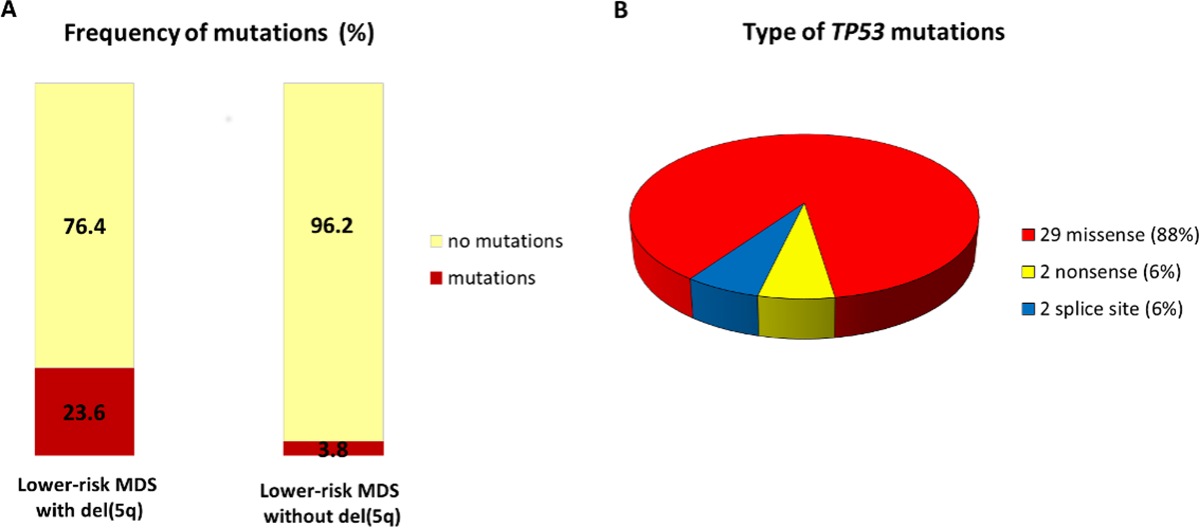
Distribution of *TP53* somatic mutations in lower-risk MDS patients (**A**) Frequency of *TP53* mutations in lower-risk MDS patients with and without del(5q). (**B**) Pie chart representing the different *TP53* mutation types detected in entire patient cohort.

In total, we identified 33 *TP53* mutations in 20 out of 154 (13.0%) patients with lower-risk MDS. Patients with del(5q) had strikingly increased prevalence of mutations compared to non-del(5q) 23.6% (17/72) vs 3.8% (3/79), respectively (Figure 1A).

Six of the patients harbored two mutations, one patient had three mutations, and one patient carried six mutations. All the mutations were located in the sequence-specific DNA-binding domain of p53 (Figure S1). A description of the individual mutations is shown in Table 2, and the profile of the type of mutations is presented in Figure 1B. The majority of the mutations were missense mutations (*N* = 29), whereas nonsense (*N* = 2) and splice site (*N* = 2) mutations were much more rare. Codons 175, 248, and 273 represent the mutational hotspots in the *TP53* gene in hemato-oncological diseases according to IARC *TP53* database (http://p53.iarc.fr/). In our cohort of patients, we detected 8 mutations in these hotspots: mutation R175H was repeatedly detected four times; R248W three times; and p.R273L once.

**Table 2:** Description of *TP53* gene mutations

Patient ID	Nucleotide position	Protein description	CD34+(%)	CD34-/MNC (%)	CD3+ cells (%)	CD14+ cells (%)	Granulocytes (%)	Cytogenetics	Interphase FISH del(5q)
624	c.517G > A	p.V173M	49.6	47.9	2.8	47.8	42.6	46, XX [16] 46, XX, del (5)(q13.2q34) [6]	89%
1125	c.581T > G	p.L194R	39.6	42.3	0.6	45.8	42.3	46, XY [15]	0%
646	c.548C > A	p.S183*	2.2	3.7	ND	1.8	2.0	46, XX [15] 46, XX, del (5)(q14q33.3) [7]	33%
306	c.733G > A c.400T > C	p.G245S p.F134L	23.9 3.5	20.5 ND	1.7 0.3	8.5 2.0	17.7 2.7	0 mitosis	28%
373	c.722C > T	p.S241F	41.9	31.1	0.0	29.2	30.2	46, XX [8] 46, XX, del (5)(q13q33) [2]	78%
272	c.734G > A	p.G245D	8.9	14.7	1.4	15.6	23.7	46, XY [15]	54%
1098	c.715A > G	p.N239D	9.0	9.5	1.5	21.7	32.9	46, XX [1] 46, XX, del (5)(q13.3q33.3)[21]	84%
1100	c.524G > A c.438G > A	p.R175H p.W146*	ND	61.4 13.7	1.1 0.0	42.0 15.4	64 25.3	Complex karyotype	ND
837	c.824G > A	p.C275Y	ND	76.8	1.1	99.1	73.2	Complex karyotype	ND
926	c.524G > A c.473G > A	p.R175H p.R158H	ND	1.1 1.9	0.0 0.0	ND	0.7 2.3	46, XX [18]	ND
1084	c.451C > T c.520A > T	p.P151S p.R174W	ND	2.1 2.5	0.1 0.2	ND	2.1 4.8	0 mitosis	19%
1095	c.659A > G c.626G > A	p.Y220C p.R209K	11.7 ND	10.0 1.7	0.1	ND	ND	46, XX[13] 46, XX, del (5)(q15q33.3) [9]	ND
141	c.375G > A	p.T125T	4.7	5.0	0	ND	3.6	46, XY [22]	0%
1043	c.818G > T	p.R273L	ND	30.8	ND	ND	ND	0 mitosis	88%
131	c.839G > T	p.R280I	30.0	18.1	ND	ND	ND	46, XY [22]	ND
112	c.395A > T	p.K132M	ND	3.0	ND	ND	ND	46, XX [4] 46, XX, del (5)(q31q33) [1]	43%
496	c.434T > C c.713G > C c.722C:T	p.L145P p.C238S p.S241F	ND	2.3 2.1 0.4	ND	ND	ND	46, XX [22]	13%
1411	c.376–2A > G c.536A > G c.742C > T c.323G > T c.524G > A c.743G > A	p.? p.H179R p.R248W p.G108V p.R175H p.R248Q	ND	3.5 34.9 2.2 1.6 1.8 1.4	ND	ND	ND	47, XX, +8 [17] 46, XX, del (5)(q13q33) [3]	50%
1207	c.742C > T	p.R248W	ND	37.1	ND	ND	ND	0 mitosis	29%
1436	c.517G > A c.524G > A	p.V173M p.R175H	ND	2.3 19.7	ND	ND	ND	46, XX [1] 46, XX, del (5)(q14q33.3) [16]	ND

Abbreviations: ND, Note Done.

**Figure 2:**
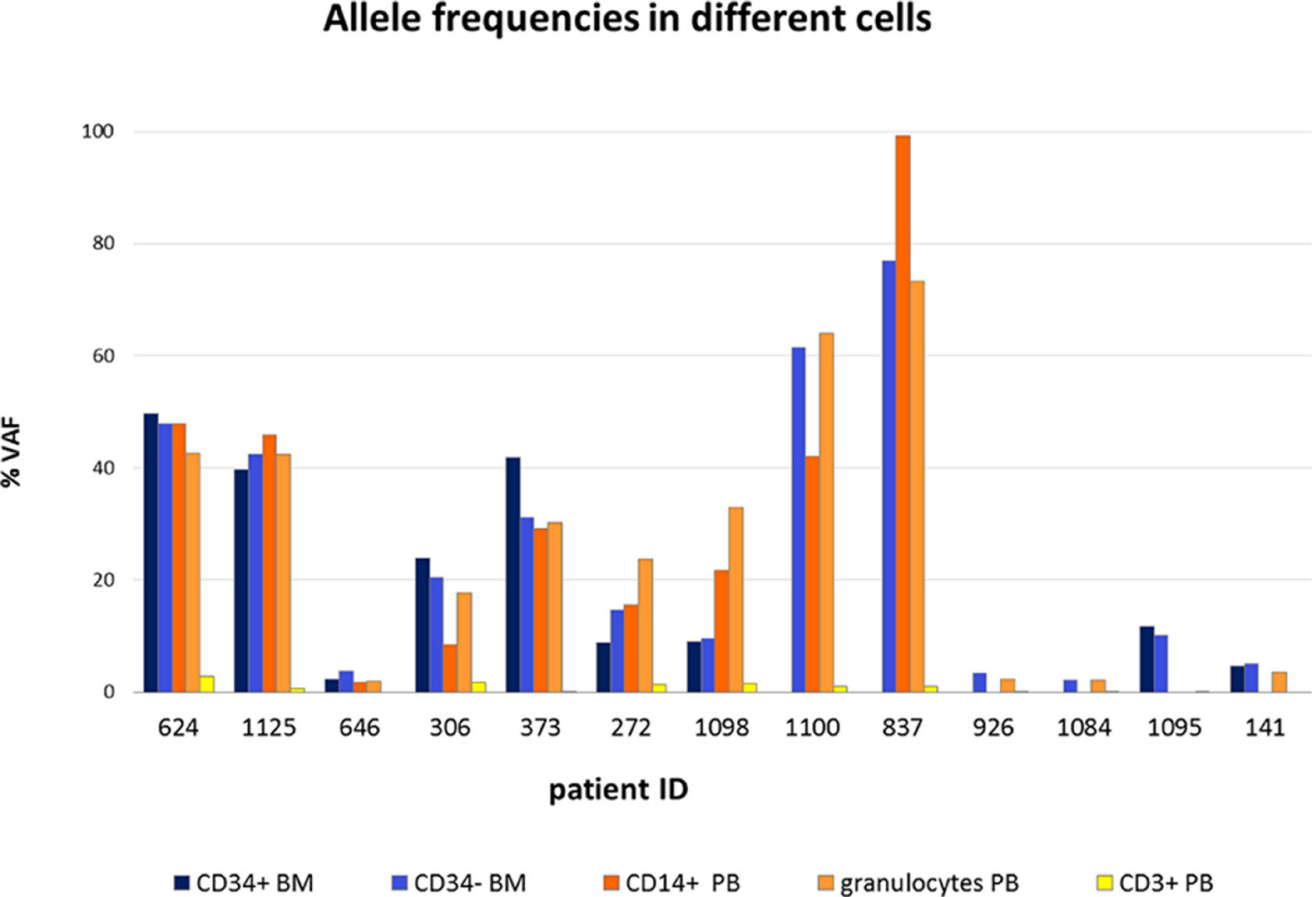
Allele frequencies of *TP53* mutations in different cell types The allele frequencies of *TP53* mutations were determined in CD34+ and CD34– cells isolated from bone marrow and CD14+ monocytes, CD3+ T lymphocytes and granulocytes isolated from peripheral blood of lower-risk MDS patients. Each column represents variant allele frequency (VAF) of *TP53* mutations in particular cell type of individual patient.

The allele frequencies of *TP53* mutations in different cell types were also determined (Figure 2) and Table 2. Pearson's rank correlation test identified significant correlations in the size of variant allele frequency (VAF) of mutations between different cell types from BM and PB (CD34+, CD34–, CD14+ and granulocytes) for a given patient (range:*r* = 0.833–0.933) except CD3+ cells (Table S1).

CD3+ T-cells were used as controls to distinguish germline and somatic mutations. A weak allele frequency of mutations was detected in these cells, which was presumably caused by contamination with mutated cells in the isolated population. The results indicate that all the detected mutations in the *TP53* gene are somatic.

### Frequency of the *TP53* allele variant and the time of mutation acquisition

The patients with detected mutations were subdivided into three groups according to the mutational burden and the time at which the mutation was first detected. The optimal cut-off of the VAF for differences in survival was determined using the R language-based web tool Cutoff Finder (http://molpath.charite.de/cutoff/) [18]. The mutation rate was 6.21% VAF (HR 11.66, 95% CI: 3.68–36.96; *p* = 1e-6) and was adopted as the best VAF cut-off value for outcome prediction (Figure S2).

The first group consisted of patients whose *TP53* mutations were already present at the time of diagnosis with mutational burden > 6% VAF. This group represented 35% of all patients with detected mutations (7 out of 20 patients). The second group constituted 35% (7 out of 20 patients) of the mutation cohort, and these patients had mutational burden < 6% VAF found at the time of diagnosis. The last group consisted of 25% of the mutation cohort (5 out of 20 patients), and these patients were negative for a *TP53* mutation at diagnosis but acquired this mutation on average 31.4 months after diagnosis (range: 18.6–48 months). Only one subject could not be evaluated because of unavailable material from the time of diagnosis. The first available sample for this patient was 8.75 years from diagnosis, when one splicing mutation (1.61% VAF) was identified.

### Univariate analysis

We performed a univariate analysis of OS and PFS to determine the prognostic impact of the following variables: age; sex; BM blasts; hemoglobin; neutrophils; platelet count; IPSS; IPSS-R risk groups; del(5q); and *TP53* mutational status. The significant predictors of OS were sex (*p* = 0.001) and platelets (*p* = 0.017). Sex (*p* = 0.001), platelet count (*p* = 0.003), and *TP53* mutational status (*p* = 0.037) were predictors of PFS. The details of the univariate analysis are provided in Table 3.

**Table 3:** Univariate analysis for overall survival (OS) and progression-free survival (PFS)

	OS			PFS		
	*P*	HR	95% CI	*P*	HR	95% CI
**Age (≥ 65 years)**	0.097	1.611	0.918–2.828	0.069	1.615	0.964–2.704
**Male sex**	**0.001**	2.612	1.492–4.574	**0.001**	2.448	1.466–4.087
**Bone marrow blasts (> 2%)**	0.211	1.416	0.821–2.441	0.391	1.242	0.757–2.039
**Hemoglobin (≥ 100 g/l)**	0.595	1.186	0.633–2.223	0.543	0.846	0.493–1.451
**Neutrophils (≥ 1.8 10^9^/l)**	0.706	1.111	0.642–1.924	0.854	1.073	0.507–2.269
**Platelets (< 100 10^9^/l)**	**0.017**	1.261	1.133–3.636	**0.003**	2.227	1.312–3.788
**Karyotype (abnormal)**	0.850	1.056	0.601–1.855	0.526	1.186	0.701–2.005
**Karyotype by del (5q)**	0.315	0.748	0.425–1.318	0.538	0.853	0.513–1.417
**IPPS (low vs. intermediate)**	0.321	1.332	0.757–2.343	0.310	1.306	0.780–2.189
**IPSS-R (very low, low vs. intermediate, high)**	0.697	1.118	0.637–1.961	0.289	1.315	0.793–2.180
***TP53* mutation**	0.086	1.767	0.922–3.388	**0.033**	2.220	1.065–4.626

Abbreviations: HR, hazard ratio; CI, confidence interval.

Bold font indicates statistically significant *P* values.

**Figure 3:**
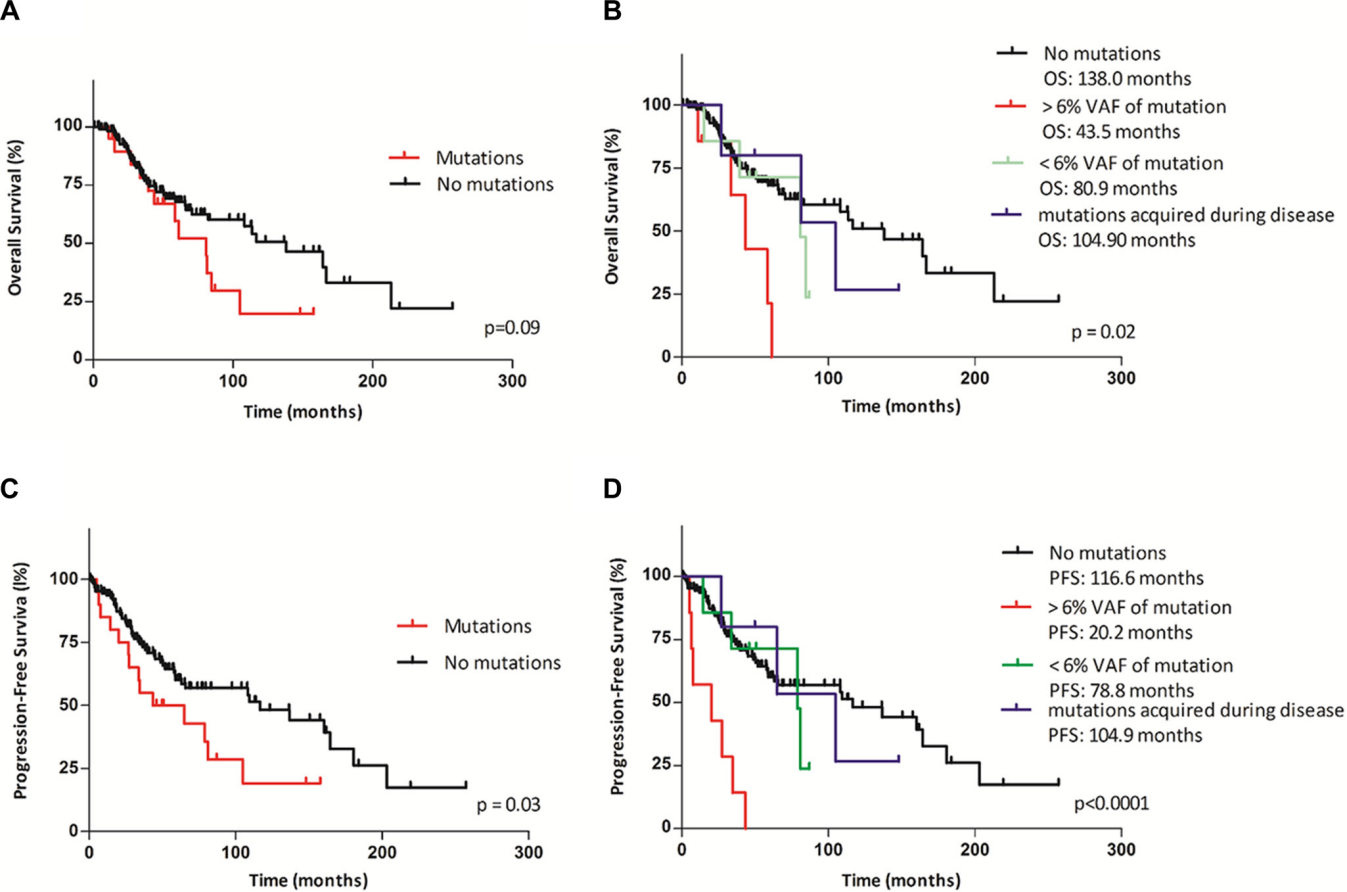
Kaplan–Meier curves of overall survival (OS) and progression-free survival (PFS) according to *TP53* mutational status and mutational burden (**A**, **C**) Comparison of OS and PFS between patients with *TP53* mutations and those with wild-type *TP53.* (**B**, **D**) Comparison of OS and PFS between patients stratified by the mutational burden and the time of mutation appearance into four groups (the first group: patients with wild-type *TP53*; the second group: patients with > 6% VAF of *TP53* mutations at the time of diagnosis; the third group: patients with < 6% VAF of *TP53* mutations at the time of diagnosis; the fourth group: patients who acquired mutations during disease. Median OS and PFS are indicated for each group).

A more detailed analysis of the prognostic impact of *TP53* mutations on OS and PFS revealed no significant differences in the OS of all MDS patients, regardless of mutation status; the median OS was 138 months in the *TP53* wild-type group and 80.9 months in the *TP53*-mutant group (*p* = 0.09) (Figure 3A). If we divided the patients into the three groups according to the mutational burden and the time at which the mutation first appeared, as described above, the OS changed significantly (Figure 3B). The first group of patients with a mutation detected at the time of diagnosis and VAF > 6% (median VAF: 43.5%; range: 23.9–76.8%) had a significantly shorter OS of 43.5 months compared to the group without a mutation (OS: 138 months; *p* = 0.003; HR 12.2, 95% CI: 2.34–63.49). The second group, which included patients with a *TP53* mutation detected at the time of diagnosis with VAF < 6% (median VAF: 1.9%; range: 0.3–4.7%), and the third group, which included patients who acquired mutations during the course of the disease (average: 31.4 months after diagnosis), did not show significant changes in OS (*p* = 0.77 and *p* = 0.99, respectively).

We next analyzed PFS according to the mutational status of the *TP53* gene using Kaplan-Meier analysis. Patients with a mutation had a significantly shorter PFS compared to those without a mutation (median: 54.2 vs. 116.6 months; *p* = 0.033; HR = 2.22, 95% CI: 1.07–4.63), regardless of the level of the mutational burden and the time of the first detection (Figure 3C). PFS changed significantly if the patients were divided into the three groups described above. PFS was dramatically reduced to 20.2 months in the subgroup of patients with mutations detected at the time of diagnosis and VAF > 6% (Figure 3D) compared to 116.6 months (*p* < 0.0001; HR = 79.46, 95% CI: 14.17–445.6) forthose without a mutation. In the group of patients with mutations identified at the time of diagnosis and VAF < 6% and in the group of patients who acquired mutations during the course of the disease, we found no significant changes in PFS (*p* = 0.53 and *p* = 0.79, respectively).

### Multivariate analysis

We performed a multivariate analysis using a Cox regression model to determine the independent impact of each variable examined for OS and PFS (Table 4). The variables included age, sex, BM blasts, hemoglobin, neutrophils, platelet count, karyotype, del(5q), IPSS, IPSS-R risk groups, and *TP53* mutational status. Multivariate analysis identified *TP53* mutational status (HR 4.389, 95% CI: 1.842–10.455; *p* = 0.001), low platelet count (HR 2.217, 95% CI: 1.052–4.673; *p* = 0.036) and male sex (HR 2.777, 95% CI: 1.404–5.494; *p* = 0.003) as significant unfavorable factors for OS. *TP53* mutational status (HR 3.743, 95% CI: 1.741–8.044; *p* = 0.001), male sex (HR 2.636, 95% CI: 1.409–4.930; *p* = 0.002) and low platelet count (HR 2.591; 95% CI: 1.330–5.051; *p* = 0.005) retained statistical significance for PFS.

**Table 4:** Multivariate Cox regression analysis for overall survival (OS) and progression-free survival (PFS)

	OS			PFS		
	*P*	HR	95% CI	*P*	HR	95% CI
**Age (≥ 65 years)**	0.122	1.028	0.993–1.063	0.115	1.023	0.994–1.053
**Male sex**	**0.003**	2.777	1.404–5.494	**0.002**	2.636	1.409–4.930
**Bone marrow blasts (> 2%)**	0.306	1.413	0.729–2.737	0.969	1.012	0.563–1.817
**Hemoglobin (≥ 100 g/l)**	0.137	1.867	0.820–4.252	0.902	1.047	0.507–2.163
**Neutrophils (≥ 1.8 10^9^/l)**	0.171	1.607	0.815–3.169	0.249	1.441	0.775–2.678
**Platelets (< 100 10^9^/l)**	**0.036**	2.217	1.052–4.673	**0.005**	2.591	1.330–5.051
**Karyotype (abnormal)**	0.110	1.993	0.855–4.645	0.091	1.958	0.897–4.274
**Karyotype by del(5q)**	0.081	0.440	0.175–1.106	0.399	0.701	0.307–1.601
**IPPS (low vs. intermediate)**	0.781	0.884	0.371–2.108	0.704	0.856	0.382–1.914
**IPSS-R (very low, low vs. intermediate, high)**	0.456	0.760	0.370–1.563	0.728	1.130	0.567–2.253
***TP53* mutation**	**0.001**	4.389	1.842–10.455	**0.001**	3.743	1.741–8.044

Abbreviations: HR, hazard ratio; CI, confidence interval.

Bold font indicates statistically significant *P* values.

### Longitudinal study of *TP53* mutations and treatment effects

The dynamics of *TP53* mutations were assessed by longitudinal ultra-deep next generation sequencing (NGS) analysis of sequential BM samples collected from 20 MDS patients.

**Figure 4:**
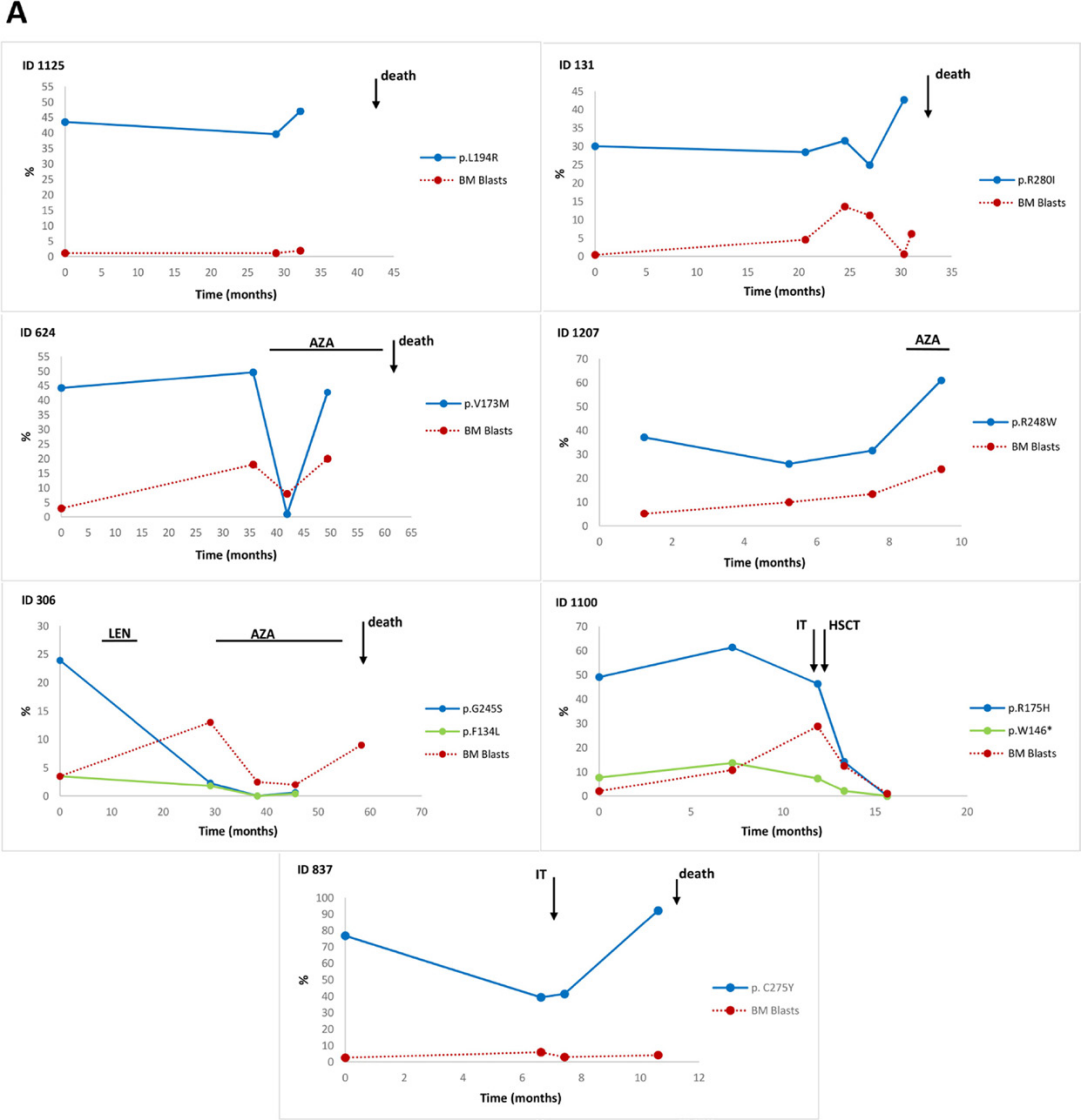

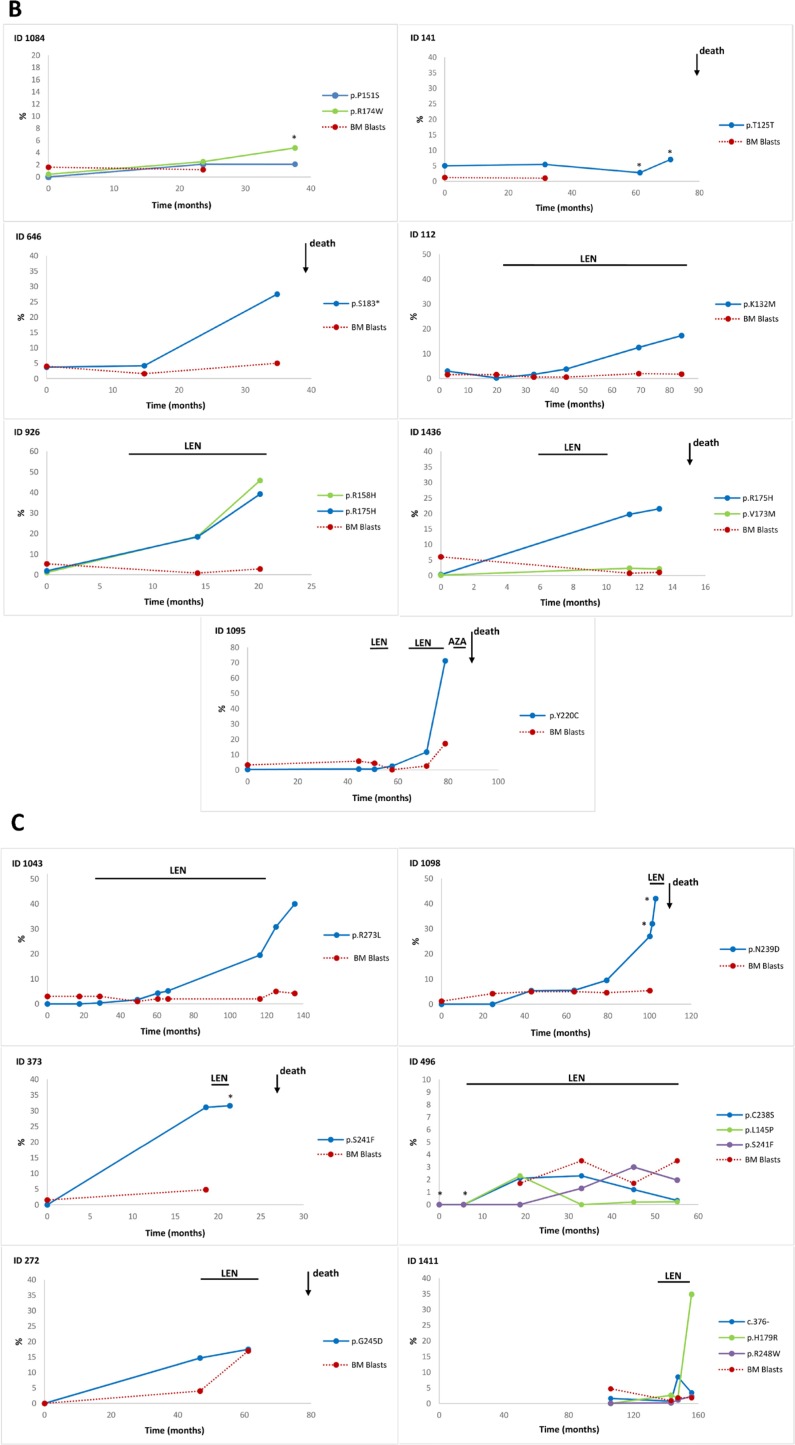
Time course of the TP53 mutant allele burden in serial follow-up samples of lower-risk MDS The frequency of *TP53* mutations during follow-up of individual patients stratified by the mutational burden and the time of mutation acquisition. (**A**) The first group of patients with VAF > 6% at the time of diagnosis; (**B**) the second group with VAF < 6% at the time of diagnosis; (**C**) the third group of patients who acquired a mutation in the course of the disease. Abbreviations: LEN, lenalidomide treatment; AZA, 5-azacytidine treatment; IT, induction therapy; HSCT, hematopoietic stem cell transplantation; *, examinations performed from peripheral blood; %, % of variant allele frequency.

The first group of patients with the worst prognosis and a high VAF at the time of diagnosis included five patients who died [four after progression (ID 837, 131, 306, 624) and one after an embolism (ID 1125)], one patients after progression on azacitidine (AZA) therapy (ID 1207), and one patient (ID 1100) who underwent of hematopoietic stem cell transplantation (HSCT) (Figure 4A).

The second group of patients with a low VAF at the time of diagnosis consisted of seven patients, four of whom died. One patient after disease progression (ID 1095), who the mutation level increased from 2.5% to 11.7% after six months of lenalidomide (LEN) treatment (71.4 months from diagnosis), and after an additional 7.4 months of treatment, this mutation burden gradually increased to 71.2%, together with the introduction of a deletion of the short arm of chromosome 17 and disease progression. Another four patients in this group (ID 646, 926, 112, and 1436) exhibited a gradual increase in mutation level to 27.5%, 39.2%, 17.3% and 21.6% after 34.8, 20.1, 84.2 and 13.2 months from diagnosis without progression, respectively, but two from these patients died. The increase in mutational burden in three from those patients (ID 926, 112, 1436) occurred after a variable period of LEN treatment (12.3, 63.8 and 4.1 months), respectively. The mutational load was repeatedly found to be below 6% in the remaining two patients (ID 1084 and 141) who have been untreated, but one died from comorbidities (Figure 4B).

The third group included five patients who acquired mutations during the disease course at a median of 31.4 months after diagnosis (range: 18.6–48 months) and one patient with low VAF at 8.75 years from diagnosis and who had no available sample from the time of diagnosis (Figure 4C). All the patients in this group were treated with LEN. Mutations were detected in four patients prior to treatment (ID 373, 272, 1098, 1411) and in two patients after treatment with LEN (ID 1043, 496). Three patients who received LEN after identification of a mutation with VAF > 6% died. Six different mutations were detected in one patient (ID 1411) after 19 months of treatment; only one mutation had a high VAF, and the remaining 5 mutations had a VAF below 5%. This patient has not experienced disease progression. The remaining two patients with mutations detected after the administration of LEN are alive. In the first patient (ID 496), two mutations were detected after one year of treatment, and the third mutation was subsequently detected; all these mutations had low-level frequencies below 5%. The second patient (ID 1043) had one mutation detected after 3 months of treatment; the mutation level gradually increased up to 40% without progression.

Additionally, we compared the OS and PFS in groups of patients who underwent treatment with LEN (treatment lasting at least four months) based on the presence of mutations. In the group of patients carrying a mutation, treatment was initiated on average 39 months after diagnosis (range: 5.9–136 months), and in patients without mutations, treatment began an average of 31 months after diagnosis (range: 2.5–135 months). As shown in Figure S3, there were no significant differences between these two groups in terms of OS (*p* = 0.18) or PFS (*p* = 0.09).

## DISCUSSION

In the present study, we collected a well characterized cohort of lower-risk MDS patients and used NGS for mutation screening of the *TP53* gene. We found that mutations in the *TP53* gene were more common in lower-risk MDS patients with del(5q) than in those without a deletion (23.6% vs. 3.8%, respectively). However, the causes of the association between mutations in *TP53* and del(5q) have not been explained yet.

In our cohort of patients, two-thirds of the mutations were detected at the time of diagnosis, and one-third of the mutations were identified during the course of the disease. Most of the mutations detected in patients with lower-risk MDS had low VAF (< 6%). These mutations can be found only exclusively using sensitive NGS technology, which is capable of identifying mutations with low abundance. Some of these identified mutations would not have been detected by Sanger sequencing because they were present at < 20% VAF.

The negative effect of small *TP53*-mutated subclones on OS has been described in chronic lymphocytic leukemia [19, 20]. In addition, Papaemmanuil et al. [21] indicated that subclonal events are likely similarly prognostically important as clonal. However, the exact prognostic impact of minor *TP53*-mutational burden in lower-risk MDS patients is not currently well understood. To assess the impact of the *TP53* mutational load on MDS survival, we determined the optimal cut-off of VAF for identifying differences in survival. Then, we divided patients into subgroups according to their *TP53* mutation abundance (above or below 6% VAF). An analysis of OS revealed significant differences among these groups. Only patients with mutations detected at the time of diagnosis with greater mutational burden (> 6% VAF) showed a significantly reduced OS compared to patients without mutations: 43.5 vs. 138 months (HR 12.18; *p* = 0.003); these patients also had a shorter PFS: 20.2 vs. 116.6 months (HR 79.5; *p* < 0.0001). Compared to patients with > 6% VAF, patients with < 6% VAF experienced longer OS (*p* = 0.06) and PFS (*p* < 0.006), but these values were not significantly different from those for patients without mutations. These findings suggest that low of *TP53*-mutational burden do not have the same unfavorable prognostic impact on OS and PFS as defects with high mutational burden in lower-risk MDS. Furthermore, we determined that *TP53* mutations (HR 3.7), male sex (HR 2.6) and low platelet count (HR 2.6) were independent predictors of PFS, and *TP53* mutations (HR 4.4), male sex (HR 2.8) and low platelet count (HR 2.2) were independent predictors of OS using multivariate analysis. Interestingly, male patients with lower-risk MDS had a worse prognosis than female patients, as described previously [22, 23]. An association between low platelet count and worse survival has also been demonstrated in patients with lower-risk MDS [24, 25].

Mutations in *TP53* may occur at different phases of malignant transformation; mutations are present in both lower-risk and higher-risk MDS, as well as in AML. *TP53* gene mutations likely contribute differently to various steps of this process [26]. *TP53* mutations may initiate malignant transformation in lower-risk MDS, induce the more aggressive growth of clones, or provide survival advantages for mutated cells, such as increased proliferation or reduced apoptosis in more advanced stages of MDS. In our patient cohort, the presence of *TP53* mutations probably represented one of the early events of malignant transformation. However, our findings suggest that lower-risk MDS patients may survive with small *TP53* subclones for several years, unlike those with higher-risk MDS and leukemias as described in some studies [17, 19, 20, and 27]. We assume that lower-risk MDS patients have more indolent forms of MDS than patients with aggressive, proliferative MDS and AML. Similarly, Jädersten et al. showed that small *TP53* subclones in low-risk MDS patients might be stable for a period of time before expanding in connection with disease progression [12]. This is in agreement with our findings that patients with a mutation frequency of less than 6% had a stable mutated clone for a long time. *TP53* mutations were found under normal conditions in small cell populations of PB in 44% healthy individuals at age 50 as part of the aging process [28]. However, an increase of the mutational burden after LEN treatment in a majority of patients (ten out of eleven patients) may reflect the selective pressure of treatment on *TP53* mutated cells. Nevertheless, this expansion of the mutated clone in response to treatment may take up to several years. Additionally, we did not detect significant differences in OS and PFS between the groups of LEN-treated patients with and without mutations.

The strong correlation between the VAFs in cells isolated from PB and BM suggests that the PB may serve as a reliable material for the detection of *TP53* gene mutations and that it is not necessary to burden patients with BM aspiration. Even mutations with low allelic frequencies (approximately 1% VAF) were detectable in both PB and BM samples. In our study, we did not see any case in which a mutation was detected in BM but not in PB. It is possible that when a mutation arises, it is first detectable in BM and later in PB, but we have not recorded such a case.

In summary, our study provides a comprehensive analysis of *TP53* mutations in different cell types and serial samples from exclusively lower-risk MDS patients. The results indicate that routine monitoring for *TP53* gene mutations in lower-risk MDS patients with del(5q) should be performed to refine the risk prediction and to enable early therapeutic intervention. The examination times for mutational analysis should be the time of diagnosis, during the course of the disease, and before starting treatment with LEN. Mutational status may be assessed in PB cells, as the results are in accordance with those from BM cells. Our data provide evidence that the *TP53* gene mutational status is an important predictor of PFS and OS, as are platelet count and sex, in lower-risk MDS patients. Particularly, the level of the mutational burden and the time at which the mutation first appears represent significant factors that determine the disease course and patient outcome.

## MATERIALS AND METHODS

### Samples and patient cohort

BM and/or PB samples were obtained from 154 patients treated at the Institute of Hematology and Blood Transfusion (*N* = 82) and the First Department of Medicine – Department of Haematology, General University Hospital and First Faculty of Medicine, Charles University (*N* = 72), Prague. Samples were obtained during routine clinical assessment. Ten age-matched healthy controls and two cord blood samples were also examined. Four out of ten control samples were isolated from BM, and the remaining six samples were isolated from PB. The median age of the controls was 69.5 years (range: 30–80 years). All the subjects provided informed consent, and the study was approved by the Local Ethics Committee. The baseline patient characteristics are listed in Table 1.

All patients were classified according to the IPSS categories at the time of sample collection, except for three patients with unavailable cytogenetics. The average follow-up interval was 56.8 months (range: 0–253 months); during that time 23.4% patients (*N* = 36) progressed (at least to RAEB-2) and 34.4% patients (*N* = 53) died. The data for 58.4% subjects (*N* = 90) were censored at the last date they were known to be alive, and 7.1% (*N* = 11) were censored at the date of HSCT. The average time of investigation of the mutational status was 32.1 months (range: 0–131 months) after diagnosis.

In total, 103 patients (66.9%) received best supportive care, 8 patients (5.2%) were treated with 5-azacytidine (Vidaza) for disease progression, 28 patients (18.2%) received LEN (Revlimid), 2 patients (1.3%) were treated with 5-azacytidine after previous treatment with LEN, and 11 patients (7.1%) underwent HSCT. LEN was administered as recommended at 10 mg/d for 21 days with a 1-week interruption. The starting dose of 10 mg was reduced to 5 mg if there was any sign of BM toxicity, such as thrombocytopenia and neutropenia. AZA was administered as recommended at 75 mg/m^2^ per day for 7 days every 28 days. Induction therapy (IT) consisted of 3 days of an anthracycline (daunorubicin 90 mg/m^2^) and 7 days of cytarabine (100 mg/m^2^).

### Cytogenetic analysis

Unstimulated BM cells were cultured for 24 hours in RPMI 1640 medium with 10% fetal calf serum. The chromosomal samples were prepared according to standard techniques with Colcemid, which included a hypotonic treatment, fixation in methanol/acetic acid, and G-banding with Wright-Giemsa stain. The karyotypes were described according to the International System of Human Cytogenetic Nomenclature (ISCN 2013) [29]. The Vysis LSI EGR1/D5S23, D5S721 Dual Color Probe (Abbott, Downers Grove, IL) was used to confirm a genetic deletion in the 5q31 region. FISH assays were performed according to the manufacturer's protocol, and at least 200 interphase nuclei were analyzed. Complex chromosomal aberrations were studied with mFISH and mBAND methods, using the 24XCyte and the XCyte color kits and an ISIS computer analysis system (MetaSystems, Altlussheim, Germany).

### Cell separation

Mononuclear cells and polymorphonuclear cells were purified by Ficoll-Hypaque density centrifugation. CD34+, CD34–, CD3+ or CD14+ cells were isolated using magnetic cell separation according to the manufacturer's recommendations (Miltenyi Biotec, Bergisch Gladbach, Germany).

### DNA extraction

The salting-out method was used to isolate DNA from separated cells. DNA was extracted from slides of BM aspirate smears using a ChargeSwitch^®^ Forensic DNA Purification Kit (Life Technologies, Carlsbad, CA) according to the manufacturer's instructions. The concentrations of DNA and RNA were assessed using a NanoDrop (Thermo Fisher Scientific, Waltham, MA) and a Qubit^®^ 2.0 Fluorometer (Life Technologies).

### DNA sequencing

Amplicon deep sequencing of *TP53* mutations (exons 4–11) was performed on a Roche 454 GS Junior system (Roche, Indianapolis, IN) using oligonucleotide primer plate assays validated according to the IRON-II (Interlaboratory Robustness Of Next generation sequencing), whose sensitivity was 1–2% in our hands. Alignment and variant calling were performed using the GS Data Analysis Software package (Roche). The mean coverage of sequenced exons was approximately 900-fold. To determine the presence of low-abundance mutant clones, the relevant exons were resequenced at a greater depth than the plate system for all exons (3000-fold). If the mutation was discovered, the mutation within the corresponding exon in different cell-enriched populations (CD34+, CD34–, CD3+, CD14+, granulocytes, and mononuclear cells) and different time points of disease were sequenced in all available samples. In some cases, DNA from BM aspirate smears was examined. A total number of 310 samples were examined for mutations. All *TP53* mutations with an allele frequency < 20% were validated by at least 2 independent ultra-deep-NGS experiments in different cell types and/or atdifferent sampling times. All *TP53* mutations with an allele frequency ≥ 20% were validated by Sanger sequencing (Applied Biosystems 3500). *TP53* mutations were annotated using the IARC TP53 database. Based on the sequencing data, we obtained VAF for each *TP53* mutation detected. VAF (also called mutant allele burden) is defined as a read count supporting the mutant base divided by the total read count at that position. A mutant allele burden of approximately 50% in regions of diploid DNA content in a homogeneous cell population indicates that all cells contain a given variant are at heterozygous state.

### Statistical analysis

The survival distributions were estimated using the Kaplan-Meier method, and the differences were compared using the log-rank test. Univariate and multivariate analyses were performed with log-rank tests and proportional hazard Cox models, respectively. The correlation of mutational burden in different cell types was measured with Spearman's rank correlation coefficient. For all analyses, *P*-values were two-tailed, and *P*-values of less than 0.05 were considered statistically significant. Analysis was conducted using IBM SPSS Statistics (IBM, NY, USA) and graphs were prepared using GraphPad Prism version 6.00 (La Jolla, CA, USA).

## SUPPLEMENTARY MATERIALS FIGURES AND TABLE


