# Supplementary material for: TP53 mutation variant allele frequency is a potential predictor for clinical outcome of patients with lower-risk myelodysplastic syndromes
Source: Oncotarget. 2016 May 6;7(24):36266–79. doi: 10.18632/oncotarget.9200 (PMC5094999; doi:10.18632/oncotarget.9200)
Supplement: Supplementary file 1 [file oncotarget-07-36266-s001.pdf]

TP53 mutation variant allele frequency is a potential predictor for clinical outcome of patients with lower-risk myelodysplastic syndromes

Supplementary Materials

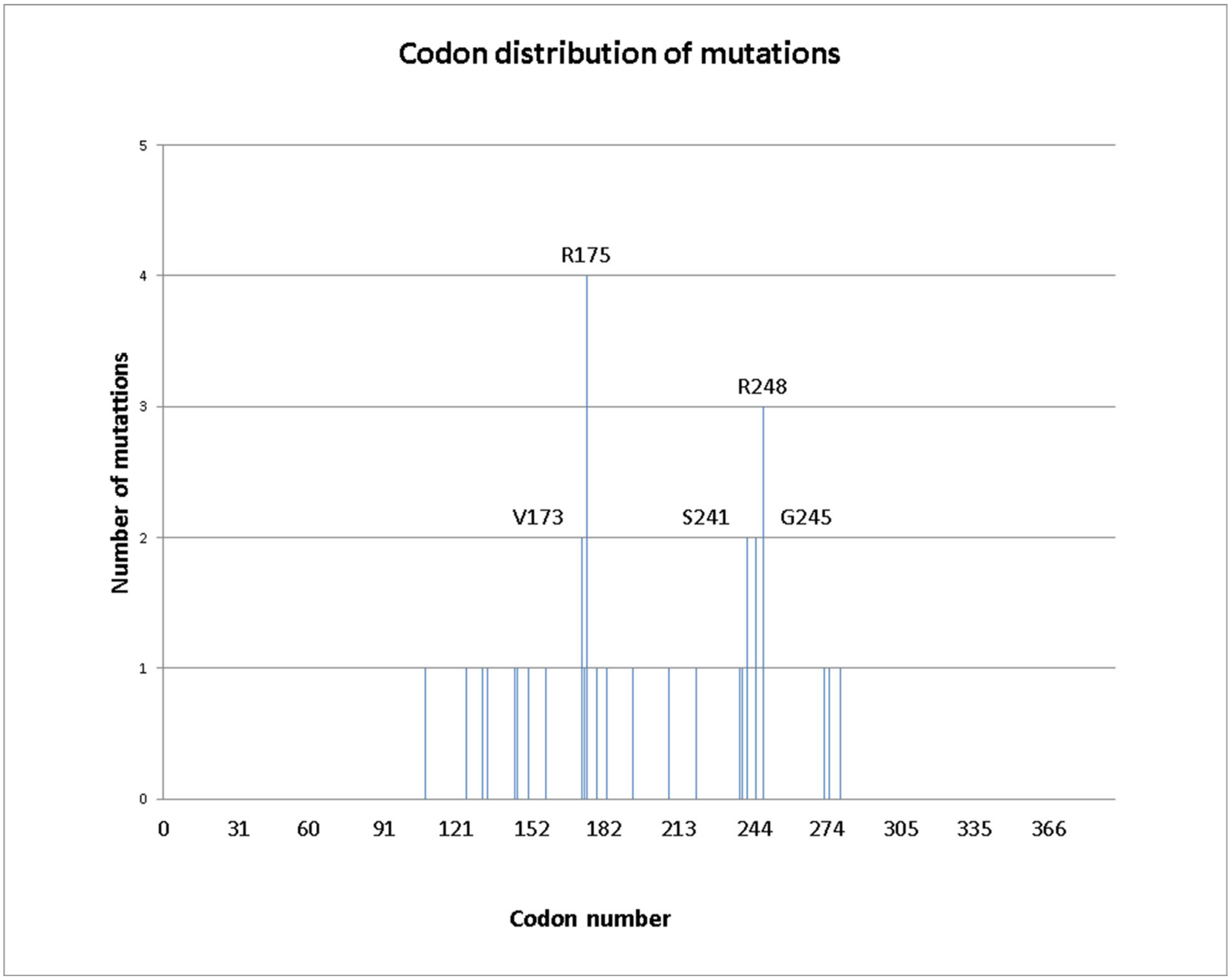

Supplementary Figure S1: Histogram displaying the position of somatic point mutations in the coding sequence of the *TP53* gene. Data from the IARC *TP53* Database. The codon distribution and number of mutations in *TP53* gene.

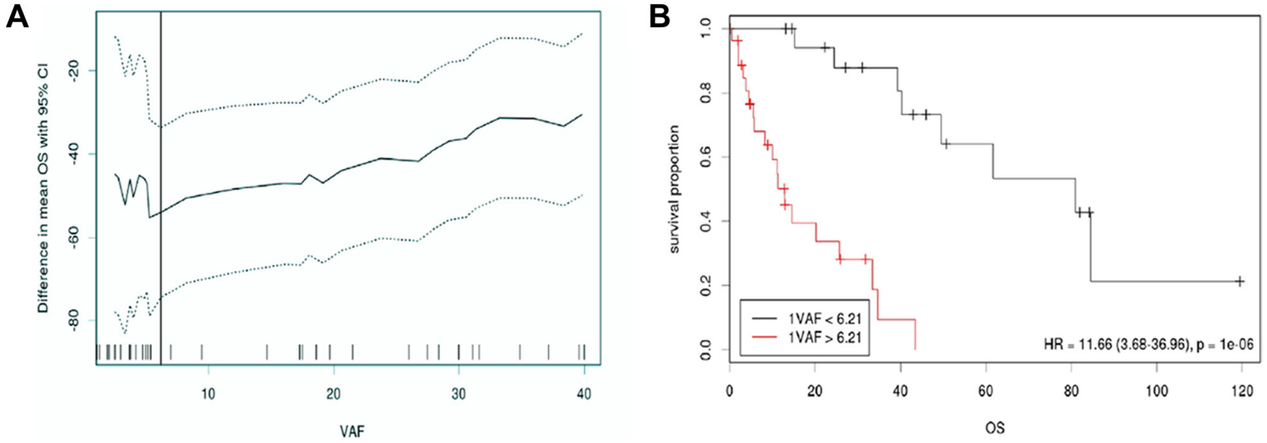

Supplementary Figure S2. Determination of the optimal cut-off of variant allele frequency (VAF) using survival significance (long-rank test) method. (**A**) Association of overall survival (OS) with VAF; (**B**) Kaplan Meier analysis of VAF using the optimal cut-off from Figure S2A.

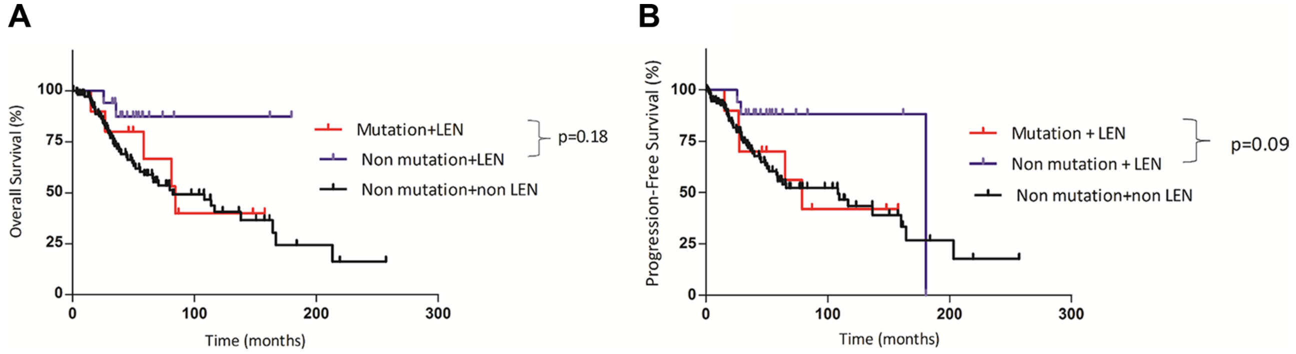

Supplementary Figure S3: Kaplan–Meier curves of overall survival (A) **and progression-free survival (B) according to *TP53* mutational status (*TP53* wild-type vs *TP53*-mutant) in patients treated with lenalidomide (LEN).** The first group: patients with *TP53* mutations and treated with LEN; the second group: patients without *TP53* mutations and treated with LEN; the third group: patients without *TP53* mutations and not treated with LEN.

Supplementary Table S1: Correlation of TP53 variant allele frequencies between different cell types

	CD34 +	CD34–	CD14+	Granulocytes	CD3+
CD34+	1	0.933**	0.857*	0.833*	0.419
CD34–	0.933**	1	0.883**	0.930**	0.541
CD14+	0.857*	0.883**	1	0.933**	–0.228
Granulocyte	0.833*	0.930**	0.933**	1	0.499
CD3+	0.419	0.541	–0.228	0.499	1

**Correlation is significant at the 0.01 level (2-tailed).

* Correlation is significant at the 0.05 level (2-tailed).
